# AD BLOOD BIOMARKERS IN DIVERSE COMMUNITY SETTINGS: A LONGITUDINAL PERSPECTIVE FROM THE ARIC STUDY

**DOI:** 10.1093/geroni/igae098.0001

**Published:** 2024-12-31

**Authors:** Priya Palta, Kevin Sullivan, Natascha Merten

**Affiliations:** Chair; Co-Chair; Discussant

## Abstract

Alzheimer’s disease and related dementias (ADRD) feature a prolonged preclinical stage spanning decades, with the transition from mid- to late-life marking the critical period for onset and accumulation of pathological brain changes that may lead to physical and cognitive disability. Identifying individuals at risk for cognitive and physical decline during preclinical stages when interventions or disease modifying treatments are more likely to be effective is needed. Blood biomarkers of ADRD pathology and neurodegeneration are promising cost-effective and non-invasive options to fill this gap. To date, however, there are limited data on temporal changes in blood biomarkers and their associations with cognitive, mobility, and neuroimaging outcomes in diverse community-based cohorts. This symposium will feature 23 years of data from the well-established community-based Atherosclerosis Risk in Communities (ARIC) Study cohort which assayed blood biomarkers of amyloid-β (Aβ)42/40, phosphorylated tau at threonine 181 (p-Tau181), neurofilament light (NfL), and glial fibrillary acidic protein (GFAP) using Quanterix Simoa assays on stored specimens at up to 3 timepoints in midlife and late-life in ~1,800 participants. This session will present findings on temporal blood biomarker changes from mid- to late-life and associated risk and protective factors of biomarker changes; mid- to late-life changes in blood biomarkers and associations with late-life neuroimaging measures of neurodegeneration, cerebrovascular disease, and amyloid deposition; and the associations of late-life blood biomarkers with prevalent and incident mobility and cognitive impairment.

